# *Rev-erbα* in the brain is essential for circadian food entrainment

**DOI:** 10.1038/srep29386

**Published:** 2016-07-06

**Authors:** Julien Delezie, Stéphanie Dumont, Cristina Sandu, Sophie Reibel, Paul Pevet, Etienne Challet

**Affiliations:** 1 Regulation of circadian clocks team, Institute of Cellular and Integrative Neurosciences, UPR3212, Centre National de la Recherche Scientifique, University of Strasbourg, France; 2 Chronobiotron, UMS3415, CNRS, University of Strasbourg, France

## Abstract

Foraging is costly in terms of time and energy. An endogenous food-entrainable system allows anticipation of predictable changes of food resources in nature. Yet the molecular mechanism that controls food anticipation in mammals remains elusive. Here we report that deletion of the clock component *Rev-erbα* impairs food entrainment in mice. *Rev-erbα* global knockout (GKO) mice subjected to restricted feeding showed reduced elevations of locomotor activity and body temperature prior to mealtime, regardless of the lighting conditions. The failure to properly anticipate food arrival was accompanied by a lack of phase-adjustment to mealtime of the clock protein PERIOD2 in the cerebellum, and by diminished expression of phosphorylated ERK 1/2 (p-ERK) during mealtime in the mediobasal hypothalamus and cerebellum. Furthermore, brain-specific knockout (BKO) mice for *Rev-erbα* display a defective suprachiasmatic clock, as evidenced by blunted daily activity under a light-dark cycle, altered free-running rhythm in constant darkness and impaired clock gene expression. Notably, brain deletion of *Rev-erbα* totally prevented food-anticipatory behaviour and thermogenesis. In response to restricted feeding, brain deletion of *Rev-erbα* impaired changes in clock gene expression in the hippocampus and cerebellum, but not in the liver. Our findings indicate that *Rev-erbα* is required for neural network-based prediction of food availability.

Light and food are the main synchronizers of the circadian multi-oscillator system. The suprachiasmatic nucleus (SCN), the master clock located in the anterior hypothalamus, is principally reset by light^1^. The SCN molecular machinery is based on transcription/translation feedback loops of clock gene/protein expression, including BMAL1 and CLOCK and their transcriptional regulators, REV-ERBα/β and RORα/β/γ^2^,^3^,^4^. Secondary oscillators, whose molecular mechanisms are close to the SCN, are also present in multiple brain regions and peripheral tissues. Timing of these circadian oscillations is coordinated by the SCN via nervous and humoral signals^1^. Feeding time can however produce phase-shifts of circadian oscillations in different organs and brain regions, independently of the SCN clock^5^,^6^,^7^,^8^.

When food access is limited to a narrow daily temporal window, animals can predict feeding time and accordingly change their activity pattern. They develop food-seeking behaviours in anticipation of mealtime, concomitant with physiological and hormonal activations^9^,^10^,^11^. Food-anticipatory activity (FAA) has the properties of a clock-controlled process and persists after SCN ablation^12^,^13^. Contrary to the SCN, the food-entrainable entity controlling FAA may not be a unique structure but rather a dispersed network of oscillators, in particular at the central level^10^,^14^,^15^,^16^,^17^,^18^,^19^.

Yet the involvement of a clock-gene dependent mechanism for food entrainment is not fully characterized, in that not all studies have agreed on the contribution of clock genes to FAA^10^,^20^,^21^,^22^,^23^. Here, we evaluated the role of *Rev-erbα*, a gear of the circadian clockwork and a regulator of glucose and lipid metabolism^4^,^24^,^25^,^26^,^27^,^28^,^29^,^30^, in the ability of mice to predict feeding time. We hypothesized that brain expression of REV-ERBα may be required to link circadian feeding cues to the central regulation of behaviour and physiology. We show that both global and brain-specific deletions of *Rev-erbα* profoundly altered the expression of food-anticipatory components. This study gives evidence that *Rev-erbα* in the central nervous system is essential to shape the 24-hour pattern of activity in conditions of limited food access.

## Results

### Food anticipation is reduced in global *Rev-erbα* KO mice in light-dark conditions

**Figure 1:**
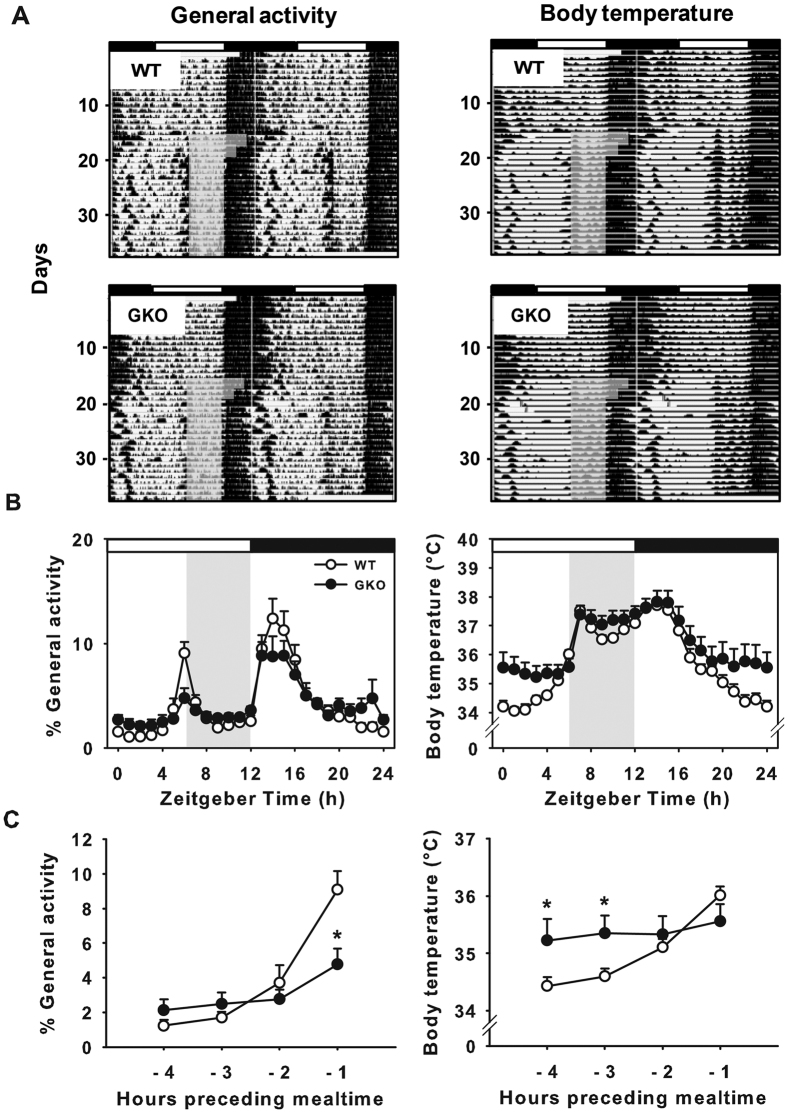
Food-anticipatory components are reduced in food-restricted *Rev-erbα* GKO mice exposed to a light-dark cycle. (**A**) Representative double-plotted actograms (left) and thermograms (right) of a wild-type (WT) mouse (upper panel) and *Rev-erbα* global knock-out (GKO) mouse (lower panel) under LD conditions with food provided *ad libitum* and then restricted to 6 h per day (grey box, food was provided from Zeitgeber (ZT) 6 to ZT12). (**B**) Normalized activity profiles and body temperature raw data representing the average of 10 days during restricted feeding conditions. Daytime and nighttime are indicated by white and black bars, respectively. The period of food access during restricted feeding is represented by the grey rectangle. Data for ZT0 are double-plotted at ZT24. (**C**) Percent of food-anticipatory (FAA) over total daily activity from 4 h to 1 h before mealtime (left) and rise in body temperature in anticipation (right). *p < 0.05 for GKO vs. WT animals (pairwise comparisons using Tukey HSD *post-hoc* test after 2-way ANOVA).

No significant differences were found between genotypes fed *ad libitum* regarding body mass and food intake, 24-h body temperature rhythm and daily general locomotor activity (Fig. 1B, as previously shown in ref. 30). We however observed that global deletion of *Rev-erb*α tended to reduce spontaneous wheel-running activity (10399 ± 2117 vs. 7174 ± 3184 revolutions/24 h in WT and GKO mice, respectively; *t*_(13)_ = 0.9, *p* = 0.4) as already found in^31^. In particular, heterogeneity in wheel-running behaviour ranged from robust rhythms to fragmented profiles, we thus excluded this parameter in most of the results presented thereafter.

**Figure 2:**
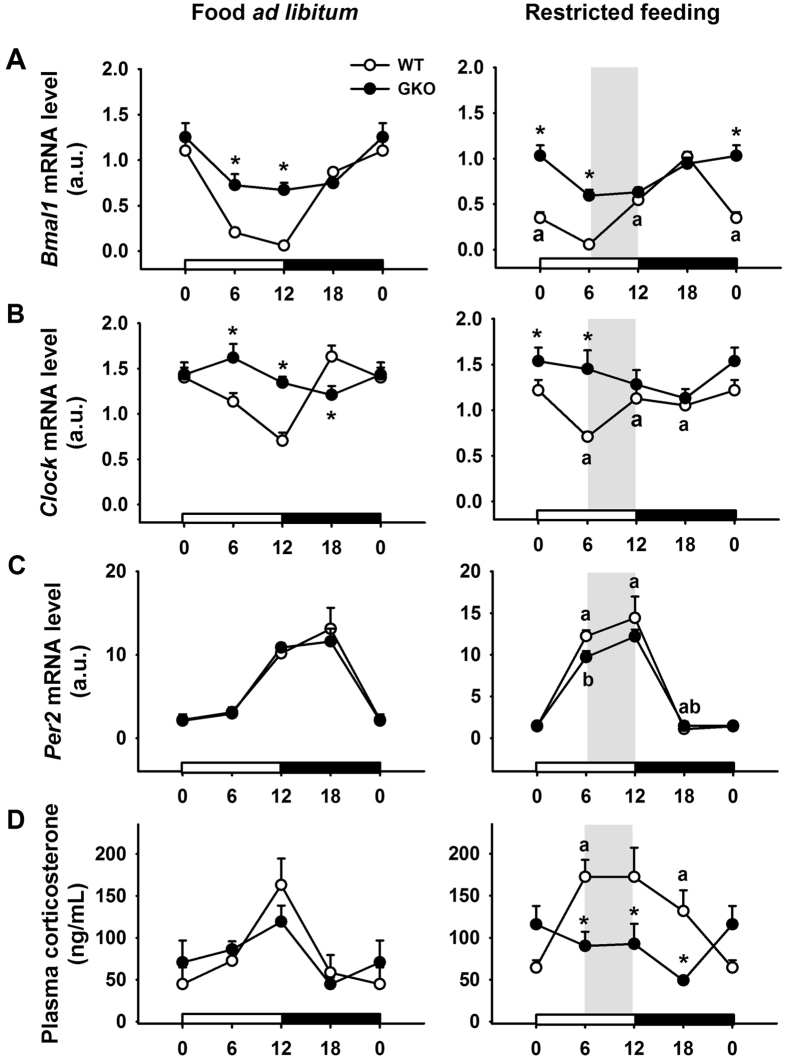
Alterations in hepatic expression of clock genes and plasma corticosterone in food-restricted *Rev-erbα* GKO mice. Expression of *Bmal1* (**A**), *Clock* (**B**), *Per2* (**C**) under conditions of food *ad libitum* (left panel) and restricted feeding (right panel). (**D**) Plasma corticosterone levels in both food *ad libitum* (left) and restricted feeding conditions (right). Daytime and nighttime are indicated by white and black rectangles, respectively, on the X axis. Food access during restricted feeding is depicted by a grey rectangle. Data for ZT0 are double-plotted. *p < 0.05 for GKO vs. WT animals at a given time-point as determined by 2-way ANOVA and Tukey HSD *post-hoc* test; ^a^p < 0.05 for restricted feeding vs. food *ad libitum* at a given time-point in wild-type (WT) mice (2-way ANOVA and Tukey post-hoc test); ^b^p < 0.05 for restricted feeding vs. food *ad libitum* at a given time-point in *Rev-erbα* global knock-out (GKO) mice (2-way ANOVA and Tukey post-hoc test). a.u., arbitrary units.

To test whether *Rev-erb*α deletion changes the ability to anticipate mealtime, global knockout (GKO) and wild-type (WT) mice were subjected to 6-h restricted feeding. As expected, WT mice expressed a strong FAA 1 h before meal access, illustrated by significant increase of general activity at that time compared to the other time-points (Effect of ZT: *F*_(3,59)_ = 20.1, *p* < 0.001; [Genotype x ZT] interaction: *F*_(3,59)_ = 5.2, *p* < 0.01 Fig. 1A–C). In contrast, GKO mice did not show a significant increase of general activity in anticipation of mealtime. The amount of general activity in GKO mice 1 h before meal access was reduced compared to WT mice (Tukey HSD *post-hoc* test, *p* < 0.05; Fig. 1A–C). Moreover, the sharp preprandial rise in body temperature seen in WT animals was not observed in GKO mice ([Genotype x ZT] interaction: *F*_(3,59)_ = 2.8, *p* < 0.05; Fig. 1B,C). Of note, both genotypes showed a similar body mass loss after one week of restricted feeding (WT = −8.1 ± 2.1 vs. GKO = −9.5 ± 3.1%; *t*_(13)_ = 0.3, *p* = 0.7.). Lastly, restricted feeding induced a 6-h phase shift of clock-related mRNA expression in the liver of WT animals, in line with previous studies^5^,^18^,^32^. Food-induced phase-adjustment of liver oscillations however also occurred in GKO mice in spite of up-regulated *Clock* and *Bmal1* expression (Fig. 2A–C). Conversely, global deletion of *Rev-erbα* abolished the anticipatory peak of corticosterone ([Genotype x ZT] interaction: *F*_(3,38)_ = 4.1 *p* = 0.01; Fig. 2D), considered as a food-anticipatory component^33^,^34^. Together, these findings indicate that behavioural and physiological components of meal anticipation are clearly blunted in GKO mice under a light-dark cycle.

### FAA in global *Rev-erbα* KO mice is altered in constant darkness

**Figure 3:**
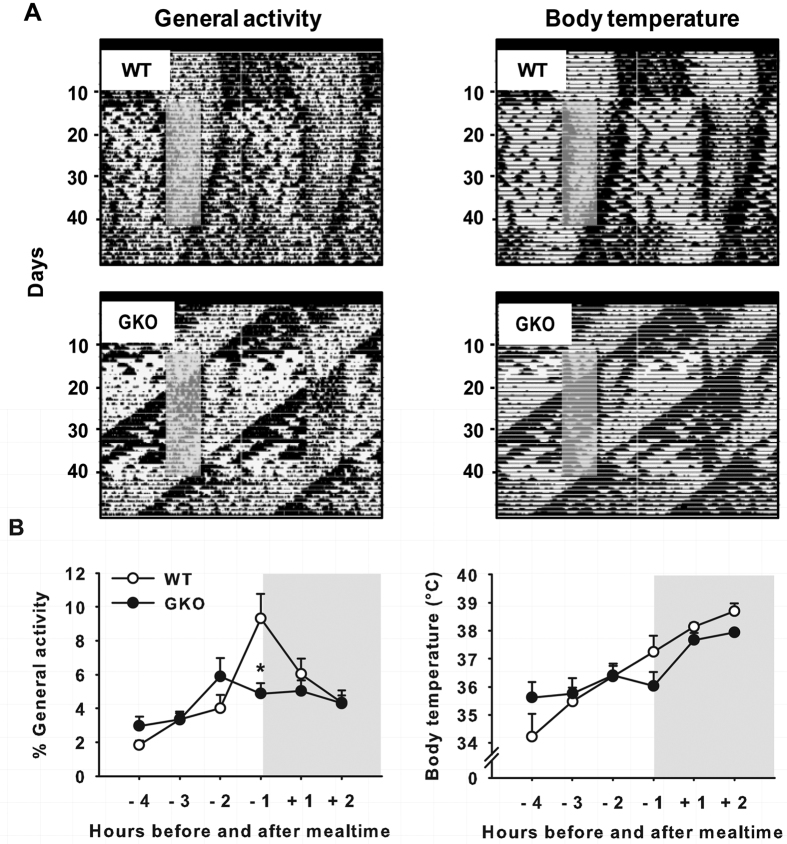
Diminished food anticipation in food-restricted *Rev-erbα* GKO mice exposed to constant darkness. (**A**) Representative double-plotted actograms (left) and thermograms (right) of a wild-type (WT) mouse (upper panels) and *Rev-erbα* global knock-out (GKO) mouse (lower panels) under conditions of constant darkness (DD) with food provided *ad libitum* and then restricted to 6 h per day (grey box, food was provided from 02:00 p.m. to 08:00 p.m.). Constant darkness is indicated by a black bar on X-axis. (**B**) Percent of food-anticipatory (FAA) over total daily activity (average of 5 days) from 4 h before mealtime and 2 h after mealtime (left) and rise in body temperature in anticipation (right). *p < 0.05 for GKO vs. WT animals (pairwise comparisons using Tukey HSD *post-hoc* test after 2-way ANOVA).

**Figure 4:**
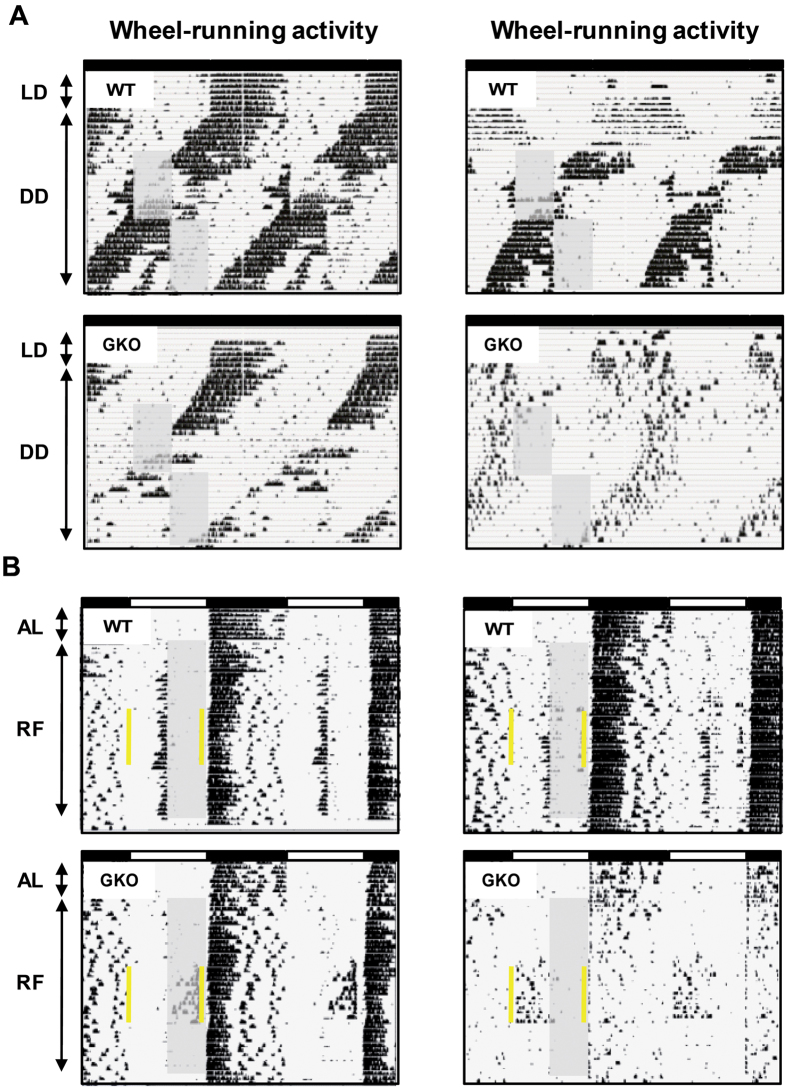
Reduced food-anticipatory activity of *Rev-erbα* GKO mice in constant darkness following shift of mealtime, and in skeleton photoperiod. (**A**) Double-plotted actograms of wheel-running activity from two wild-type (WT) mice (upper panel) and two *Rev-erbα* global knock-out (GKO) mice (lower panel). Animals are in constant dark conditions (DD, indicated by the above dark bar). A few days in *ad libitum* (LD) feeding conditions are illustrated before transfer to DD. During restricted feeding, food access is indicated by the grey rectangle. After a first period of food restriction at a specific circadian phase, mealtime was shifted (i.e., 6-h phase-delayed). (**B**) Double-plotted actograms of wheel-running activity from two WT mice (upper panel) and two GKO mice (lower panel) exposed to skeleton photoperiod. Note that the 12-h light and dark phases are represented respectively by white and black bars above the actograms, and that the two 1-h light pulses are indicated by yellow lines. A few days of *ad libitum* (AL) feeding conditions is illustrated before food restriction (RF, grey rectangle).

To avoid a potential masking effect of light known to attenuate activity in nocturnal animals^35^, which can account for the reduced food anticipation in *Rev-erbα*-deficient mice, we exposed WT and GKO mice to constant dark (DD) conditions or to skeleton photoperiod. In DD conditions with animals fed *ad libitum*, the global deletion of *Rev-erbα* shortened the free-running period (WT = 24.1 ± 0.1 h vs. GKO = 23.2 ± 0.1 h; *t*_(9)_ = 13.9, *p* < 0.001; as in ref. 4). Furthermore, mealtime was shifted (i.e., 6-h phase-delayed) to investigate how FAA reappears at a new feeding time. In both DD conditions (with or without shift of mealtime) and skeleton photoperiod, we did not detect more FAA in GKO mice than in light-dark (LD) conditions (Figs 3A,B and 4A,B).

Hence, regardless of the lighting conditions, the time of the day when food was provided, global deletion of *Rev-erbα* markedly reduces FAA.

### Feeding time differently affects PER2 and p-ERK expressions in the cerebellum of global *Rev-erbα* KO mice

**Figure 5:**
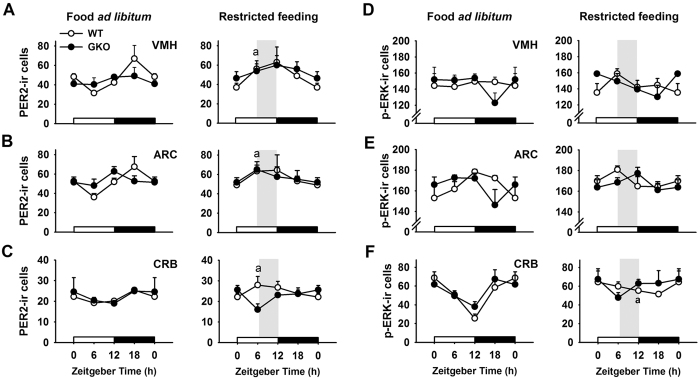
Differential changes of PER2 and p-ERK expression in the hypothalamus and cerebellum of *Rev-erbα* GKO and WT mice challenged with restricted feeding. (**A**–**C**) PER2 and (**D**–**F**) p-ERK immunoreactive (ir) cells in ventromedial (VMH) and arcuate (ARC) hypothalamic nuclei, and cerebellum (CRB) of mice under conditions of food *ad libitum* or exposed to restricted feeding schedules. Note that staining intensity was taken into consideration for all hypothalamic nuclei; while for the cerebellum, the number of labeled Purkinje cells was counted. ^a^p < 0.05 for restricted feeding vs. food *ad libitum* at a given time-point in wild-type (WT) mice (2-way ANOVA and Tukey post-hoc test). Data for ZT0 are double-plotted.

A number of extra-SCN oscillators sensitive to feeding cues could be part of the food-entrainable network^8^,^14^,^36^. We therefore investigated PER2 expression and used the phosphorylated form of the Extracellular signal Regulated Kinases I/II (p-ERK) as a marker of neuronal activation in response to restricted feeding in hypothalamic nuclei, including dorsomedial (DMH), arcuate (ARC), suprachiasmatic (SCN), ventromedial (VMH) and paraventricular (PVN) nuclei, and the cerebellum. When food was provided *ad libitum*, PER2 expression was rhythmic in all and most evaluated structures in WT and GKO mice, respectively (Supplemental Table S1). Similar timing was found in the SCN in both genotypes. There was a trend for phase-advanced peak in the ARC and PVN of GKO mice (and possibly also in VMH and cerebellum) compared to controls (Fig. 5A–C; Supplemental Table S1). Expression of p-ERK was rhythmic in the cerebellum with close timing in GKO and WT mice fed *ad libitum* (Supplemental Table S2). In the ARC, p-ERK expression was rhythmic in both genotypes, while in other hypothalamic regions (VMH, DMH, PVN and SCN), no significant cosinor regression was detected (Fig. 5D,E; Supplemental Table S2). Furthermore, except for the cerebellum, the daily pattern of p-ERK expression did not exactly match between WT and GKO, with apparent phase-changes and/or dampened amplitude (Fig. 5F; Supplemental Table S2).

Besides the SCN insensitive to mealtime, adaptation of PER2 oscillations to restricted feeding was observed in most hypothalamic nuclei (ARC, VMH and PVN) of WT mice, as shown by an earlier PER2 expression that increased during food access (Fig. 5A,B, Supplemental Table S1). Close phase-advances were found for ARC and PVN (and a trend for VMH) of GKO mice. By contrast, PER2 expression in the cerebellum was markedly phase-advanced in food-restricted WT mice, but not in GKO mice (Fig. 5C, Supplemental Table S1). In response to daytime feeding, p-ERK expression was phase-advanced in the ARC of WT mice, while this change was hardly detectable in GKO mice (Fig. 5D,E, Supplemental Table S2). In the cerebellum, p-ERK expression in WT animals was clearly up-regulated before and during food access, while in GKO mice, the daily trough of p-ERK expression occurred prior to mealtime (Fig. 5F).

In sum, neuronal activation during food anticipation is less marked in the hypothalamus and cerebellum of GKO mice, which may contribute to their reduced FAA. Furthermore, phase-adjustment of the circadian clockwork to feeding time does not require the expression of *Rev-erbα* in hypothalamic and hepatic oscillators, whereas it seems indispensable for cerebellar clock adjustment.

### Circadian phenotype of brain specific KO for *Rev-erbα* fed *ad libitum*

**Figure 6:**
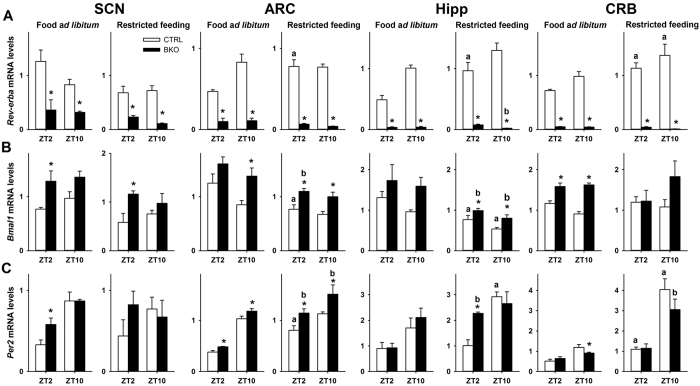
Clock gene mRNA levels are altered in the brain of *Rev-erbα* BKO mice. (**A**) *Rev-erbα,* (**B**) *Bmal1* and (**C**) *Per2* mRNA levels in different brain regions from Control *NesCre-Rev-erbαΔ*/+ (CTRL) and *NesCre-Rev-erbαΔ*/− (BKO) mice fed *ad libitum*. SCN: suprachiasmatic nucleus, ARC: arcuate nucleus, Hipp: hippocampus, CRB: cerebellum, ZT: Zeitgeber Time. *p < 0.05 for GKO vs. WT animals at a given time-point as determined by 2-way ANOVA and Tukey HSD *post-hoc* test; ^a^p < 0.05 for restricted feeding vs. food *ad libitum* at a given time-point in wild-type (WT) mice (2-way ANOVA and Tukey post-hoc test); ^b^p < 0.05 for restricted feeding vs. food *ad libitum* at a given time-point in *Rev-erbα* brain-specific knock-out (BKO) mice (2-way ANOVA and Tukey post-hoc test).

To further evaluate the central contribution of *Rev-erbα* to food entrainment, we generated brain-specific KO mice. Moreover, the use of conditional KO mice may circumvent potential confounding effects of global clock gene deletion on whole-body energy homeostasis and therefore on food entrainment (e.g., refs 37, 38, 39). The *NesCre*-induced recombination of the floxed *Rev-erbα* allele was confirmed from multiple brain regions of *NesCre-Rev-erbαΔ*/− mice (noted BKO below) by qPCR analysis, in comparison to *NesCre-Rev-erb*Δ/+ mice (noted CTRL thereafter). We observed a nearly non-existent expression of the *Rev-erbα* transcript in the cerebellum (Effect of Genotype, *F*_(1,21)_ = 16.1, *p* < 0.001). In addition, we performed exploratory analyses in other brain regions. Very efficient *NesCre*-driven recombination was found in the hippocampus (Effect of Genotype, *F*_(1,18)_ = 286.9, *p* < 0.001; Fig. 6A), nucleus accumbens, striatum, and motor cortex (data not shown). Down-regulation of *Rev-erbα* expression was less strong in the SCN and ARC of BKO mice, although *Rev-erbα* mRNA levels were still very significantly reduced and did not cycle between ZT2 and ZT10 (Effect of Genotype: *F*_(1,18)_ = 24.0 and *F*_(1,18)_ = 102.9, respectively in the SCN and ARC, *p* < 0.001 for both structures; Fig. 6A). Of note, one mouse heterozygous for *NesCre* and carrying both a KO and a floxed allele as confirmed by pre-weaning and post-death genotyping was excluded from the analysis, since *Rev-erbα* mRNA levels were normally expressed in the SCN of this mouse, which furthermore demonstrated a normal circadian phenotype (data not shown). These data indicate that the *NesCre*-driven recombination is not fully consistent amongst brain regions, in line with previous studies that used the same strategy^37^. As a result of *Rev-erbα* knockdown in the brain, we detected a significant elevation of the *Bmal1* transcript at both time points, regardless of the brain regions considered (Effect of Genotype, *p* < 0.05 in the cerebellum, SCN, ARC and hippocampus. For details of Tukey HSD *post-hoc* tests, see Fig. 6B). Conversely, *Per2* mRNA levels were less affected in most brain nuclei (Effect of Genotype: *p *> 0.05 in the cerebellum, SCN and hippocampus; and significant in the ARC: *F*_(1,18)_ = 18.9, *p* < 0.01 Fig. 6C), while we observed reduced amplitude of *Per2* between both time points in several brain regions of BKO mice, including the cerebellum (Effect of ZT: *F*_(1,18)_ = 38.1, *p* < 0.01; [Genotype x ZT] interaction: *F*_(1,18)_ = 7.6, *p* = 0.01; Fig. 6C).

**Figure 7:**
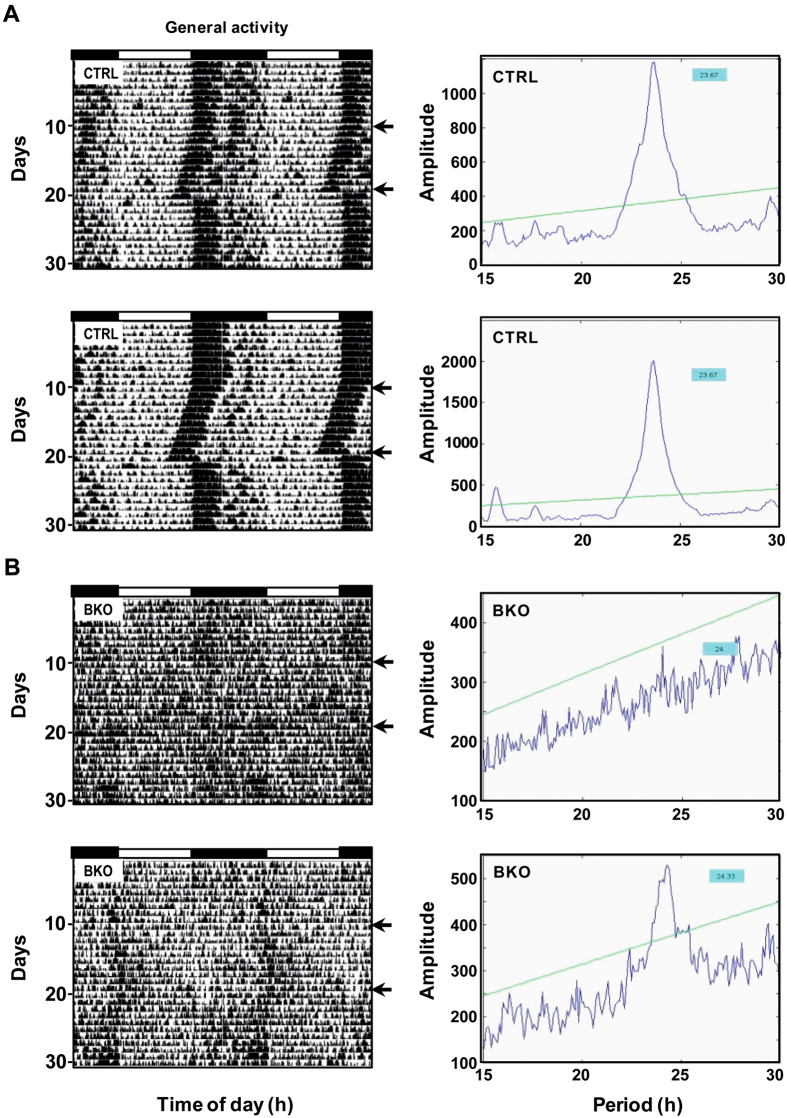
Circadian activity profiles of *Rev-erbα* BKO mice are markedly disturbed under light-dark cycle and constant darkness. Left panels: (**A**) Representative double-plotted actograms of two free-fed control *NesCre-Rev-erbαΔ*/+ (CTRL) and (**B**) two *NesCre-Rev-erbαΔ*/− (BKO) mice under light-dark (LD) conditions and then exposed to constant darkness (DD; delimited by horizontal black arrows) for 10 days. Right panels: Corresponding χ^2^ periodograms from (**A**) 2 CTRL and (**B**) 2 BKO mice.

In LD conditions with food *ad libitum*, brain deletion of *Rev-erbα* did not affect body mass (21.6 ± 0.9 vs. 23.7 ± 1.2 g in BKO and CTRL mice, respectively; *t*_(11)_ = 1.4, *p* = 0.18). All control groups (*NesCre-Rev-erb*Δ/+, *Rev-erbα*flox/+, *Rev-erbα*flox/−) showed similar circadian organization and levels of both daily general locomotor and wheel-running activities (data not shown). We however observed a virtual absence of wheel use in BKO mice, thus precluding reliable comparison of wheel-running activity with the other genotypes (data not shown). The amplitude of day-night changes in general locomotor activity was drastically reduced without brain expression of *Rev-erbα.* In some animals, activity was rather fragmented and bursts of activity occurred mainly at the day-night/night-day transitions (Fig. 7A,B). The circadian general locomotor activity in BKO mice was further altered in DD conditions with a strong heterogeneity: three BKO mice were behaviourally arrhythmic, one exhibited a shorter and the other two had a longer free-running period compared to CTRL mice (Fig. 7A,B). These changes highlight major changes in the light-entrainable suprachiasmatic clock.

### Diminished *Rev-erbα* expression in the brain accounts for altered food entrainment

**Figure 8:**
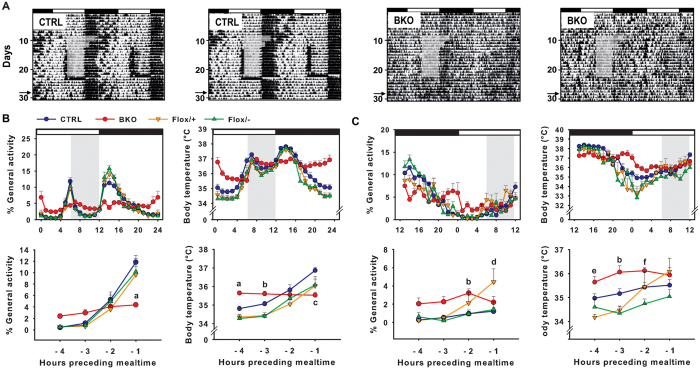
Food-anticipatory components are not expressed in food-restricted *Rev-erbα* BKO mice exposed to a light-dark cycle. (**A**) Representative double-plotted actograms of control *NesCre-Rev-erbαΔ*/+ (CTRL) animals and *NesCre-Rev-erbαΔ*/− (BKO) mice under LD conditions with food provided *ad libitum* and then restricted to 6 h per day (grey box, food was provided from ZT6 to ZT12). Note that on the last day of restricted feeding (day 24), food was given 6 h later (i.e., ZT12) than before. Thereafter, food was provided *ad libitum* except from day 28 (ZT12) to day 29 (ZT12) during which no food was given (“fasting test”, see arrow on Y axis). (**B,C**, upper panels) Normalized activity profiles and body temperature raw data representing the average of 10 days during restricted feeding conditions (**B**) and fasting test (**C**) in *NesCre-Rev-erbαΔ*/+ (CTRL, blue circle), *NesCre-Rev-erbαΔ*/− (BKO, red circle), *Rev-erbα*flox/+ (Flox/+, orange down-pointing triangle) and *Rev-erbα*flox/− (Flox/−, green up-pointing triangle) mice. Daytime and nighttime are indicated by white and black bars above the graphs, respectively. The period of food access during RF is represented by the grey rectangle. (**B**,**C**, lower panels). Statistical analysis (2-way ANOVA) was performed on percent of FAA over total daily activity and body temperature data from 4 h to 1 h before expected mealtime. ^a^*p* < 0.05 for BKO vs. other groups, ^b^*p* < 0.05 for BKO vs. Flox/+ and Flox/1, ^c^*p* < 0.05 for CTRL vs. other groups, ^d^*p* < 0.05 for Flox/+ vs. other groups, ^e^*p* < 0.05 for BKO vs. Flox/+, and ^f^*p* < 0.05 for BKO vs. Flox/−.

To determine whether *Rev-erbα* in the brain is required for circadian food entrainment, we challenged BKO mice with 6-h restricted feeding in LD conditions. All food-restricted control mice rapidly developed FAA 1 to 2 h prior to mealtime (Fig. 8A,B), the peak of FAA 1 h before food access representing about 10 to 12% of daily total activity in all control groups. By contrast, BKO mice did not readily express behavioural activation 1 h before food arrival, FAA being hardly detectable above their mean level of daily activity ([Genotype x ZT] interaction: *F*_(9,83)_ = 6.9, *p* < 0.001; For details of Tukey HSD *post-hoc* tests, see Fig. 8B). In accordance with behavioural data, the peak of thermogenesis in anticipation was totally abolished in BKO mice (Effect of genotype: *F*_(3,83)_ = 6.2, *p* < 0.01; [Genotype x ZT] interaction: *F*_(9,83)_ = 7.9, *p* < 0.001; Fig. 8B). Moreover, like GKO mice, food-restricted BKO mice were able to maintain body temperature levels close to those measured in *ad libitum* conditions. During the 6-h food access, the daily amount of food eaten was smaller in BKO (*NesCre-Rev-erb*Δ/−) mice compared to CTRL (*NesCre-Rev-erb*Δ/+) animals (2.9 ± 0.2 g vs 3.8 ± 0.1 g, respectively; *t*_(11)_ = 3.9, *p* < 0.01), while body mass during and at the end of the 2 wks of restricted feeding was not significantly different between the two genotypes (19.4 ± 1.0 vs. 22.4 ± 1.0 g, respectively; *t*_(11)_ = 2.1, *p* = 0.06) as was body mass loss (−10.0 ± 2.3% vs. −5.3 ± 0.9; respectively; *t*_(11)_ = 2.0, *p* = 0.07).

Interestingly, in food-restricted CTRL mice, *Rev-erbα* expression was up-regulated at ZT2 in the ARC, Hipp and CRB, while *Rev-erbα* levels were as low as during *ad libitum* conditions in brain areas of BKO mice (for details, see Fig. 6A). Expression of *Bmal1* and *Per2* was not affected in the SCN of food-restricted BKO and CTRL mice in comparison with conditions of food *ad libitum* (Fig. 6B,C) and *Bmal1* was still up-regulated in the SCN of food-restricted BKO animals (Effect of Genotype: *F*_(1,13)_ =  43.1, *p* < 0.001). Besides *Rev-erbα*, morning and evening expression of *Per2* and/or *Bmal1* was also altered in the ARC, hippocampus and CRB of CTRL mice in response to restricted feeding (Fig. 6). In the ARC, levels of *Per2* and *Bmal1* were up-regulated in food-restricted BKO mice as compared to CTRL animals (Effect of Genotype: *F*_(1,15)_ > 12, *p* < 0.01 for both genes), while phase-changes were close between genotypes ([Genotype x ZT] interaction: *F*_(1,15)_ < 0.1, *p* > 0.8) for both genes). In the hippocampus, *Bmal1* mRNA levels were down-regulated in food-restricted mice (Fig. 6), albeit at higher levels in BKO as compared to CTRL mice (Effect of Genotype: *F*_(1,16)_ =  11.1, *p* < 0.01). *Per2* expression in the hippocampus was differentially affected by mealtime according to the genotype ([Genotype x ZT] interaction: *F*_(1,16)_ = 7.7, *p* = 0.01): *Per2* mRNA levels were increased in the morning only in BKO and in the evening only in CTRL mice. Finally, in addition to increased *Rev-erbα* levels in response to mealtime in the cerebellum of CTRL mice, *Per2* expression was also up-regulated, indicating phase-sensitivity to mealtime. In the cerebellum of BKO mice, the morning levels of *Per2* expression was similar between restricted feeding and *ad libitum* food, suggesting impaired phase-adjustment of the cerebellar clock to mealtime (Fig. 6C). In contrast, except for *Per2* expression in BKO animals that was more diminished in the morning, changes in clock gene expression in the liver of food-restricted mice were close between BKO and CTRL groups (Supplemental Fig. 1), indicating that the phase-adjustment of the liver clock to mealtime was essentially unaltered in BKO mice.

We also tested the reappearance of FAA: food-restricted mice were transferred back to conditions with *ad libitum* food for 5 days and then challenged with a 24-h fast. Among the fasted control groups, *Rev-erbα* flox/+ mice (i.e., mice that possess both a WT allele and a non-recombined floxed allele) showed a strong increase in locomotor activity before expected food access while the progressive elevation of FAA was much slighter in the other controls (Effect of genotype: *F*_(3,83)_ = 6.3, *p* < 0.01; [Genotype x ZT] interaction: *F*_(9,83)_ = 2.7, *p* = 0.01; Fig. 8C). On the other hand, a rise in body temperature before expected mealtime was observed in all control groups (Effect of genotype: *F*_(3,83)_ = 6.1, *p* < 0.01; [Genotype x ZT] interaction: *F*_(9,83)_ = 2.9, *p* < 0.01; Fig. 8C). Notably, neither FAA nor food-anticipatory thermogenesis could be observed in fasted BKO mice (Fig. 8C). Overall, the lack of behavioural and physiological components of food anticipation in a brain-specific KO model reveals that central *Rev-erbα* expression is crucial for proper food entrainment.

## Discussion

The role of clock genes in the food-entrainable network has been assessed by challenging clock-compromised mice with time-restricted feeding. Some studies suggest that circadian food anticipation relies on a clock gene-dependent mechanism that at least involves the *Period1-3, Bmal1, Cry1-2* and *Npas2* genes^23^,^32^,^37^,^39^,^40^,^41^,^42^,^43^. Others indicate that contribution of clock genes to food entrainment is mild^44^,^45^, or even non-significant^21^,^22^,^46^. Here we give evidence that central *Rev-erbα* expression is necessary for an animal to predict the time of food availability.

In conditions of limited food access, global *Rev-erbα* deletion induces a significant reduction of FAA in LD conditions. This dampened FAA was further confirmed in DD conditions or skeleton photoperiod. In accordance with behavioural results, the food-anticipatory thermogenesis was nearly absent in GKO mice. By contrast, the postprandial peak of body temperature matched between WT and GKO mice, indicating similar food processing. In addition, we observed similar body mass values and no significant reduction or delay in food intake between food-restricted WT and GKO animals (data not shown), thus excluding poor health status as a cause of reduced food anticipation. Lastly, corticosterone levels were not elevated in anticipation of daytime feeding in GKO mice. Hence, the overall altered food-entrained physiology of GKO mice supports an essential role for *Rev-erbα* in food entrainment.

The liver clock is sensitive to feeding cues^32^ and *Rev-erbα* may play a role in that regard^47^. Phase-adjustment of hepatic clock actors to feeding time was however preserved in GKO mice. This indicates that disrupted FAA is not the consequence of impaired liver clock entrainment to scheduled feeding. Of note, the expression of clock-related genes in the liver of *Per2* mutant mice is also shifted in response to daytime feeding^32^. It is thus likely that the liver clock is robust to clock perturbations on the long term and may rely on metabolic cues to adjust the phase of circadian mRNA expression in conditions of time-restricted food access.

PER2 expression has been shown to respond to feeding time in hypothalamic oscillators^8^. Our results show that PER2 expression in the hypothalamic nuclei studied was not significantly different between both genotypes, either fed *ad libitum* or challenged with restricted feeding. Therefore, the diminished capability of food entrainment in GKO mice is not correlated with altered PER2 cycling in hypothalamic areas. Among hypothalamic oscillators, several studies have been focused on the DMH, thought to be part of the food-entrainable network^15^,^19^,^48^, but see also^49^,^50^). Here we did not detect sizeable PER2 immunostaining in the DMH, as previously reported^8^. Together, these data indicate that *Rev-erbα* may be dispensable for adjustment of hypothalamic clock gene oscillations as in the liver, although these results do not rule out a role for hypothalamic nuclei in food entrainment.

Increased expression of c-FOS has been observed before and/or after mealtime in several hypothalamic nuclei, including the DMH and the VMH^15^,^36^,^51^. By studying the activation marker p-ERK, we showed for the first time that its daily pattern of expression was adjusted to feeding time in all hypothalamic nuclei of WT animals. On the contrary, p-ERK expression was affected by global *Rev-erbα* deletion in both feeding conditions and no peak of p-ERK expression was detected in GKO in anticipation of mealtime. This indicates that feeding/homeostatic signals are not similarly integrated in hypothalamic areas of GKO mice compared to WT mice, while PER2 phase-adjustment to scheduled feeding remains unaffected in the absence of *Rev-erbα* and the consecutive alteration of p-ERK activation. Distinct cellular pathways, therefore, may be involved in the entrainment of brain oscillators and in the induction of immediate early genes in response to feeding-related cues.

On the other hand, additional brain areas may be essential to drive FAA. In support to that view, brain-specific KO of *Bmal1*^37^, but not forebrain-specific deletion^38^, reduces FAA, suggesting that midbrain or hindbrain structures may modulate FAA. The cerebellum has recently been shown to harbor a circadian oscillator sensitive to feeding schedules. Furthermore, genetic and pharmacological impairments of the cerebellar function disrupt FAA in mice^14^. In this context, we determined the expression pattern of both PER2 and p-ERK proteins in the cerebellum of WT and GKO mice. We found no phase-adjustment of PER2 oscillations to feeding time, as well as a diminution of p-ERK expression in anticipation of feeding in GKO mice. Noteworthy, *Per2* mRNA is not increased in anticipation to mealtime in *Grid2*^*ho*/*ho*^ mice that have genetic cerebellar deficits, and which do not express FAA^14^. It is thus likely that altered PER2 expression in the cerebellum of food-restricted *Rev-erbα* GKO mice is somewhat related to their defect to show strong food-anticipatory components. In the brain network of food-entrainable clocks, additional evidence is given here that the cerebellum may be important to integrate homeostatic signals and/or to drive FAA.

To further evaluate the central contribution of brain expression of *Rev-erbα* to food entrainment, we used a conditional gene KO approach. *Rev-erbα* expression was prevented in the nervous system by using the *NesCre* driver. As a result, *Rev-erbα* mRNA expression was barely detectable and did not cycle in the brain of BKO mice fed *ad libitum* or food-restricted, while *Bmal1* transcript was constitutively up-regulated at the two studied time points. This however did not severely affect *Per2* cycling in brain regions, in spite of reduced amplitude of *Per2* transcripts between morning and evening in the SCN and cerebellum of BKO mice. In LD conditions with food provided *ad libitum*, brain deletion of *Rev-erbα* induced a more drastic reduction of both general locomotor and wheel-running activities than global *Rev-erbα* deletion did. Likewise, BKO mice showed a further altered circadian phenotype than that displayed by GKO mice, which is likely due to the conditional gene knockout strategy used in this study that imposes temporal and regional restriction of *Rev-erbα* deletion. Moreover, these changes somehow contrast with the shorter free-running period of GKO mice for *Rev-erbα* (our results and ref. 4), but are reminiscent of the very weak circadian rhythmicity of double GKO for *Rev-erbα* and *Rev-erbβ*^29^.

Similarly to global deletion of *Rev-erbα*, faint expression of this gene restricted to the nervous system disrupts food entrainment. BKO mice did not show substantial FAA during the 2 weeks of food restriction. This result indicates that circadian (i.e., self-sustained) properties of FAA are altered in the absence of central *Rev-erbα* expression. In addition, our qPCR data in CTRL mice show that *Rev-erbα* expression highly responds to daytime restricted feeding in different brain nuclei, except the SCN. Furthermore, brain *Rev-erbα* deletion impaired changes in clock gene expression in response to limited food access in the hippocampus and cerebellum, but not in the ARC and liver. Therefore, our results indicate that *Rev-erbα* is essential to couple the nutritional state and the clock in some brain nuclei and support the view that the food-entrainable circadian system relies on a complex population of genes and oscillators with probably distinct roles.

Lastly, we observed that while control mice decreased their body temperature during food withdrawal, BKO animals maintained body temperature values close to those observed in conditions of food *ad libitum*. The absence of phase- and amplitude-adjustment of the body temperature rhythm in BKO mice may indicate that restricted feeding does not trigger adaptive mechanisms to reduce energy expenditure when food becomes scarce, to the same degree as in control mice. Interestingly, higher values of body temperature were observed not only in food-restricted GKO mice (present results), but also in 24-h food deprived or cold-exposed GKO animals^30^,^52^. The relative hyperthermic state of GKO and BKO could thus point to a role for *Rev-erbα* in the central regulation of energy expenditure. To what extent possible changes in thermoregulatory responses could have affected expression of FAA in GKO and BKO needs to be further investigated.

Together, the present findings demonstrate that *Rev-erbα* in the brain is required not only for proper functioning of the light-entrainable master clock, but also to integrate feeding cues and adjust circadian behaviour and physiology to environmental conditions, critical for animal survival.

## Methods

### Animals and housing conditions

The founders *Rev-erbα* heterozygous mice kindly provided by Prof. Ueli Schibler (University of Geneva, Switzerland) have been rederived on C57BL6J background^30^. The *Rev-erbα* deletion strategy is described in^4^. *NestinCre* transgenic mice were obtained from The Jackson Laboratory (#003771). The conditional *Rev-erbα* KO model was established at the Mouse Clinical Institute (Strasbourg, France) in the framework of the European EUMODIC consortium^53^. To create a conditional allele of *Rev-erbα*/*Nr1d1* (MGI:2444210), an MCI proprietary vector containing flipped Neomycin resistance cassette was used, resulting in step1 plasmid. The floxed 0.6 kb fragment encompassing exons 3 and 4 was amplified by PCR and subcloned in step2 plasmid. In parallel, a 3.7 kb fragment corresponding to the 5’ homology arm and 2.9 kb fragment corresponding to the 3’ homology arms were amplified by PCR and subcloned in step2 plasmid to generate the final targeting construct (see Fig. S2). The linearized construct was electroporated in 129S2/SvPas mouse embryonic stem (ES) cells. After selection, targeted clones were identified by PCR using external primers and further confirmed by Southern blot with 5’ and 3’ external probes. Two positive ES clones were injected into C57BL/6N blastocysts, and male chimaeras derived gave germline transmission. The conditional mouse model was then backcrossed in C57BL/6J background. To produce brain-specific KO mice, we crossed animals with a conditional *Rev-erbα* allele with mice carrying a *Cre* recombinase transgene under the control of the rat *Nestin* promoter. To improve the efficiency of deletion of *Reverbα* alleles, we used the same strategy described as in ref. 37 (see Fig. S3). Note that a significant decrease of *Rev-erbα* expression was detected by qPCR analysis in liver tissue of BKO mice fed *ad libitum*. This mild effect, however, did not affect at all the day-night expression of *Bmal1* and *Per2*, suggesting a fully functional circadian clock in the liver (Fig. S1).

Mice of both sexes (sex ratio about 1:1) were bred in a pathogen-free facility (Chronobiotron platform, UMS 3415, CNRS and University of Strasbourg) in a temperature-controlled room (22 ± 1 °C) under 12 h light and 12 h dark (LD 12:12) conditions unless otherwise stated. Regular chow (SAFE 105, Augy, France) and water were provided *ad libitum,* except during the periods of restricted feeding. Access to food during restricted feeding was set automatically by the Fasting Plan system (Intellibio, Seichamps, France). Two- to 5-month old mice were individually housed in transparent plastic cages equipped with a running wheel (12.5 cm in diameter). Body mass and food intake were determined weekly. All experiments were performed in accordance with the NIH Guide for the Care and Use of Laboratory Animals (1996), the French National Law (implementing the European Union Directive 2010/63/EU) and approved by the Regional Ethical Committee of Strasbourg for Animal Experimentation (CREMEAS).

### Locomotor activity and body temperature recordings

Mice were implanted i.p. under constant gaseous anesthesia (oxygen with a constant rate at 0.2 L/min and isoflurane at 2%) with a small transponder (G2 E-Mitter, MiniMitter, Bend, Oregon, USA) recording gross locomotor activity (general activity hereafter) and core body temperature every 5 min. A PC-based acquisition system (VitalView, MiniMitter) recorded the aforementioned parameters plus the wheel-running activity 24 h a day.

### Experimental design

#### Global KO (GKO) – LD conditions

WT and GKO mice (n = 7–8/genotype) were fed *ad libitum* for 2–3 weeks and then exposed to restricted feeding schedules in which food availability was progressively reduced to 6 h per day, from ZT6 to ZT12 (ZT refers to Zeitgeber Time, ZT0 and ZT12 defining time of lights-on and lights-off, respectively) for up to 3 weeks. Of note, LD experiment was reproduced with an additional series of mice for the metabolic and mRNA/protein expression analyses presented in this study.

#### Global KO – DD conditions

WT and GKO mice (n = 4–6/genotype) were transferred for several weeks to constant darkness (DD) and exposed to temporal restricted feeding during which the food access was limited to 6 h per day for 3 weeks (from 02:00 p.m. to 08:00 p.m.).

#### Global KO – DD conditions and shift of mealtime

Mice (n = 4/genotype) were placed in DD conditions and fed *ad libitum* and then challenged with a restricted feeding paradigm in which food was given for 6 h from 02:00 p.m. to 08:00 p.m for 4 weeks. After a first period of food restriction at a specific circadian phase, mealtime was shifted (i.e., 6-h phase-delayed) to investigate how FAA reappears at a new feeding time.

#### Global KO – Skeleton photoperiod

Mice (n = 4/genotype) were exposed to skeleton photoperiod (i.e., 1 h light-pulse at the beginning of the resting period and 1 h light-pulse at the end of the resting period) and challenged with a 6-h restricted feeding schedule.

#### Brain-specific KO (BKO)

*NestinCre*+;*Rev-erbα*Δ/+ (CTRL, n = 7), *NestinCre*+;*Rev-erbα*Δ/− (BKO, n = 7; note that one mouse was excluded *a posteriori* from the analysis due to poor recombination leading to normal levels of *Rev-erbα* in brain; see results), *Rev-erbα*flox/+ (n = 4) and *Rev-erbα*flox/− (n = 4) were fed *ad libitum* for 2 weeks in LD conditions and then challenged with a restricted feeding schedule in LD conditions in which food availability was reduced to 6 h per day, from ZT6 to ZT12, for up to 2 weeks. Then mice were fed *ad libitum* for 5 days, and were fasted for 24 h (from ZT12 to ZT12) to test the reappearance of FAA. The animals fed *ad libitum* were also exposed to DD conditions to evaluate their endogenous period. CTRL and BKO mice fed *ad libitum* were sampled in the morning (ZT0-ZT2) together with additional *Rev-erbα*flox/+ and *Rev-erbα*flox/− mice fed *ad libitum* sampled in the evening (ZT10-ZT12) to check for recombination in the brain and liver and possible day-night variations in gene expression. Another set of CTRL and BKO mice were exposed to a restricted feeding schedule under LD for 2 weeks, as reported above. Then, brains and livers were sampled in in the morning (ZT0-ZT2) or in the evening (ZT10-ZT12).

### Tissue and blood collections

GKO and WT mice from LD experiments, either with food *ad libitum* or restricted feeding conditions, were sacrificed at ZT0, ZT6, ZT12 and ZT18. Briefly, mice were injected with a lethal dose of pentobarbital, a blood sample was taken intracardially and two pieces of liver were cut and immediately flash-frozen in liquid nitrogen. Then animals were perfused transcardially with 50 mL PBS 1X (Phosphate-buffered saline, pH 7.2) and 50 mL PFA buffer (4% paraformaldehyde in 0.1 M phosphate buffer, pH 7.4). Brains were removed and post-fixed in 4% PFA for 24 h at 4 °C and cryoprotected successively for 24 h at 4 °C into 10, 20 and 30% sucrose solution in 0.1 M PB. Brains were then frozen in isopentane at −40 °C and then stored at −80 °C. *Rev-erb*Δ/− mice and their controls were sacrificed as described above. Brain were quickly removed and frozen in isopentane cooled with liquid nitrogen, and liver samples were cut and immediately flash-frozen in liquid nitrogen and stored at −80 °C.

### Hormonal measurements

Plasma corticosterone was measured using an EIA kit (AC-14F1, ImmunoDiognosticSystems, Paris, France). The limit of sensitivity was 0.55 ng·mL^−1^.

### Immunohistochemistry

Coronal frozen brain sections (30 μm thick) of the hypothalamus and the cerebellum of WT and GKO mice were made using a cryostat (CM3050, Leica Biosystems). Free-floating sections were rinsed in PBS 1X and incubated in a solution of 3% H_2_O_2_ (Sigma-Aldrich) in PBS for 30 min at room temperature. Sections were then rinsed in PBS, and incubated for 2 h in a blocking solution containing 10% goat serum in PBS with 0.3% Tween 20 in PBS. Then sections were incubated in the primary antibody solution (in PBS + 0.3% Tween 20 + 10% goat serum) for 24 h with gentle agitation at 4 °C. We used a rabbit polyclonal anti-PER2 (1:2000; Alpha Diagnostic International, Cat. PER21-A; #869900A1) and a rabbit anti-p44/42 MAPK (1:20000; Cell Signaling #4370). Sections were then rinsed in PBS with 0.05% Tween 20 and incubated for 2 h at 4 °C with a biotinylated anti-rabbit IgG made in goat (Vectastain ABC peroxidase kit PK6101), diluted 1:500 with 0.3% Tween 20 in PBS on a plate shaker at 4 °C. Thereafter, sections were rinsed in PBS + 0.05% Tween 20 and incubated for 1 h at room temperature with an avidin-biotin-peroxidase complex (1:250; Vectastain Kit, PK6101; Vector Laboratories) in PBS + 0.05% Tween 20. Next, sections were rinsed in PBS, and incubated with 3,3′-diaminobenzidine (0.5 mg/mL; Sigma) with 0.015% H_2_O_2_ in H_2_O. Thereafter, sections were rinsed with PBS, wet-mounted onto gel-coated slides, dehydrated through a series of alcohol, soaked in toluene, and coverslipped with Eukitt (Sigma-Aldrich). Photomicrographs were taken on Leica DMRB microscope (Leica Microsystems) with an Olympus DP50 digital camera (Olympus France). The intensity and number of immunoreactive cells in hypothalamic nuclei and the Purkinje layer of the cerebellum, respectively, were determined using NIH ImageJ software (Rasband, W.S., U. S. National Institutes of Health, Bethesda MD, USA). The average intensity or cell numbers was determined, as far as possible, from three brain sections per animal. The number of animals/genotype/ZT was comprised between 3 and 6. SCN staining served as an internal control to evaluate the reliability of our immunohistochemistry experiments.

### mRNA extraction and Quantitative Real-Time PCR of cerebellum and brain punches

Frozen brains from BKO mice and their controls were placed in a cryostat, cerebellum were separated from the rest of the brain, homogenized in lysis buffer supplemented with *β*-mercaptoethanol and total RNA was immediately extracted according to the manufacturer’s protocol (RNeasy Mini Kit, Qiagen). With the remaining brain tissue, serial coronal sections (200 μm thick) were made in the cryostat and immediately placed in RNAlater (Ambion). Brain nuclei were identified under a stereomicroscope with a mouse brain atlas and dissected with the brain punch tissue set (Leica Biosystems). Punches were put in lysis buffer and total RNA extracted using the RNeasy Micro Kit (Qiagen). RNA quantity and quality were measured using NanoDrop ND-1000 Spectrophotometer (NanoDrop Technologies) and the Bioanalyzer (Agilent RNA 6000 Pico kit, Agilent Technologies), respectively. cDNAs were synthesized from 200 ng (cerebellum) and 10–100 ng (brain punches) of total RNA using the SuperScript III Kit (Invitrogen). Quantitative Real-time PCR was performed and analyzed using an Applied Biosystems 7300 Real-time PCR System with 1X TaqMan Gene Expression Master Mix (Applied Biosystems), 1X TaqMan Gene Expression Assay (Applied Biosystems, see references below) and 1 μL of cDNA in a total volume of 20 μL. PCR conditions were 10 min at 95 °C followed by 40 cycles of 15 s at 95 °C, 1 min at 60 °C. PCR reactions were done in duplicate. Relative expression levels were determined using the comparative ΔC_T_ method to normalize target gene mRNA to *Tbp*. The following TaqMan Gene Expression Assays were used: *Bmal1* (Mm00500226_m1), *Per2* (Mm00478113_m1), *Nr1d1* (exon 4–5; Mm00520711_g1) and *Tbp* (Mm00446971_m1).

### mRNA extraction and Quantitative Real-Time PCR in liver

Pieces of frozen livers from GKO mice and their control littermates, or BKO and CTRL mice were homogenized in lysis buffer supplemented with β-mercaptoethanol and total RNA was extracted according to the manufacturer’s protocol (Absolutely RNA Miniprep Kit, Stratagene, Agilent technologies). RNA quantity was measured using NanoDrop ND-1000 Spectrophotometer (NanoDrop Technologies). cDNAs were synthesized from 1 μg of total RNA using the High Capacity RNA-to-cDNA Kit (Applied Biosystems). Quantitative Real-time PCR was performed as above. Relative expression levels were determined using the comparative ΔCT method to normalize target gene mRNA *to β-actin* and *Tbp* for GKO and BKO samples, respectively. The following TaqMan Gene Expression Assays were used: *β-actin* (Mm01205647_g1), *Bmal1* (Arnt1, Mm00500226_m1), *Clock* (Mm00455950_m1), *Tbp* (Mm00446971_m1), and *Per2* (Mm00478113_m1).

### Analysis of locomotor activity and temperature data

Daily rhythms of general activity and body temperature were analyzed using a Clocklab software (Actimetrics, Evanston, IL, USA) associated to MatLab (MathWorks, France). Locomotor activity data were double-plotted in actograms using the Clocklab percentile format in all the figures. Mean activity profiles were quantified every 1 h during the last 10 days of food *ad libitum* and restricted feeding conditions in the LD experiments while for the DD experiments only 5 days were considered in the analysis to avoid an overlap between the FAA and the activity controlled by the SCN. For comparison purpose, data points are expressed as a percentage of total daily activity. To determine the free-running period of animals in DD conditions, the χ^2^ periodogram was used. Note that alpha was set at 0.01 and that 10 days were considered.

### Statistical analysis

All values are expressed as mean ± SEM. Normality and homogeneity of variance were assessed with Kolmogorov–Smirnov Lilliefors test and Levene’s test, respectively. Data non-normal and/or heteroscedastic were subjected to logarithmic transformation before analysis. Alpha was set at 0.05. Data were analyzed either with Student’s t-test or two-way ANOVA followed by Tukey HSD *post-hoc* analysis when applicable. Statistical analyses were performed with Statistica version 10 (StatSoft, Maisons-Alfort, France). For assessing daily rhythmicity of PER2 and p-ERK levels in brain structures, we used a cosinor analysis to determine mean level, amplitude and acrophase of the rhythm with SigmaPlot software (Systat software Inc., San Jose, CA, USA). Data were fitted to the following regression: [y = a + b·cos(2·π·(x − c)/24)] where a is the mean level, b the amplitude, and c the acrophase of the rhythm. Cosinor regressions were considered as significant only when the 3 fitted parameters had *p* ≤ 0.05.

## Additional Information

**How to cite this article**: Delezie, J. *et al.* *Rev-erbα* in the brain is essential for circadian food entrainment. *Sci. Rep.* **6**, 29386; doi: 10.1038/srep29386 (2016).

## Supplementary Material

Supplementary Information
