# Supplementary material for: Rev-erbα in the brain is essential for circadian food entrainment
Source: Sci Rep. 2016 Jul 6;6:29386. doi: 10.1038/srep29386 (PMC4933951; doi:10.1038/srep29386)
Supplement: Supplementary Information [file srep29386-s1.pdf]

***Rev-erbα* in the brain is essential for circadian food entrainment**

Julien Delezie, Stéphanie Dumont, Cristina Sandu, Sophie Reibel, Paul Pevet, & Etienne Challet[1*]

Supplemental Information

Figure S1: Synchronization of the liver clock by mealtime in *Rev-erbα* GKO mice

Figure S2: Conditional knock-out strategy

Figure S3: Breeding scheme of *Rev-erbα* GKO mice

Supplemental Tables

Figure S1: Synchronization of the liver clock by mealtime in *Rev-erbα* BKO mice

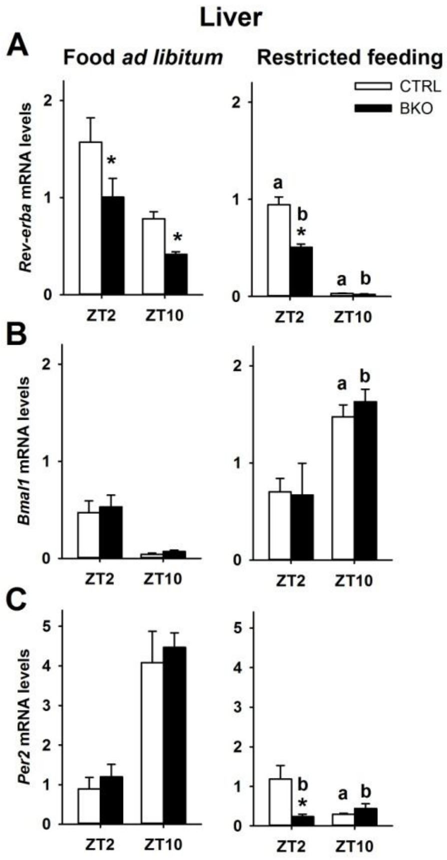

Expression of *Rev-erbα* (A), *Bmal1* (B), *Per2* (C) under conditions of food *ad libitum* (left panel) and restricted feeding (right panel). * $p<0.05$ for BKO vs. WT animals at a given time-point as determined by 2-way ANOVA and Tukey HSD *post-hoc* test; ^{a} $p<0.05$ for restricted feeding vs. food *ad libitum* at a given time-point in wild-type (WT) mice (2-way ANOVA and Tukey post-hoc test); ^{b} $p<0.05$ for restricted feeding vs. food *ad libitum* at a given time-point in *Rev-erbα* brain-specific knock-out (BKO) mice (2-way ANOVA and Tukey post-hoc test). ZT: Zeitgeber Time.

Figure S2: Conditional knock-out strategy

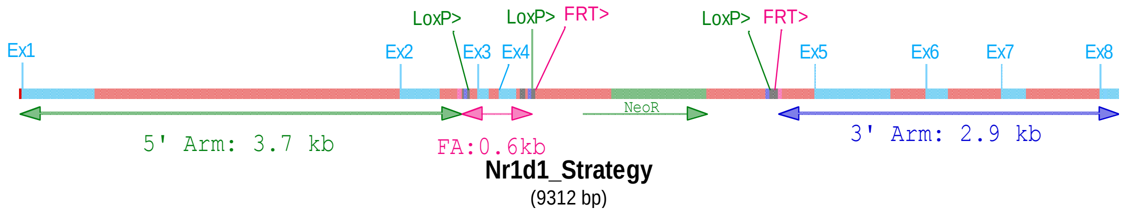

Strategy chosen: flox of exons 3 and 4.
Nr1d1 gene (also named *RevErb*α) is a member of the nuclear receptor family. Additional information on this gene can be accessed at:
http://www.informatics.jax.org/javawi2/servlet/WIFetch?page=markerDetail&key=85194
LoxP and FRT sites are indicated in green and pink, respectively. *Mus musculus* sequences are indicated in uppercase. Exogenous sequences are marked in lowercase. The targeting vector was generated in 129Sv/Pas (Mouse Clinical Institute, Strasbourg, France and European EUMODIC consortium).

Figure S3: Breeding scheme of *Rev-erbα* BKO mice

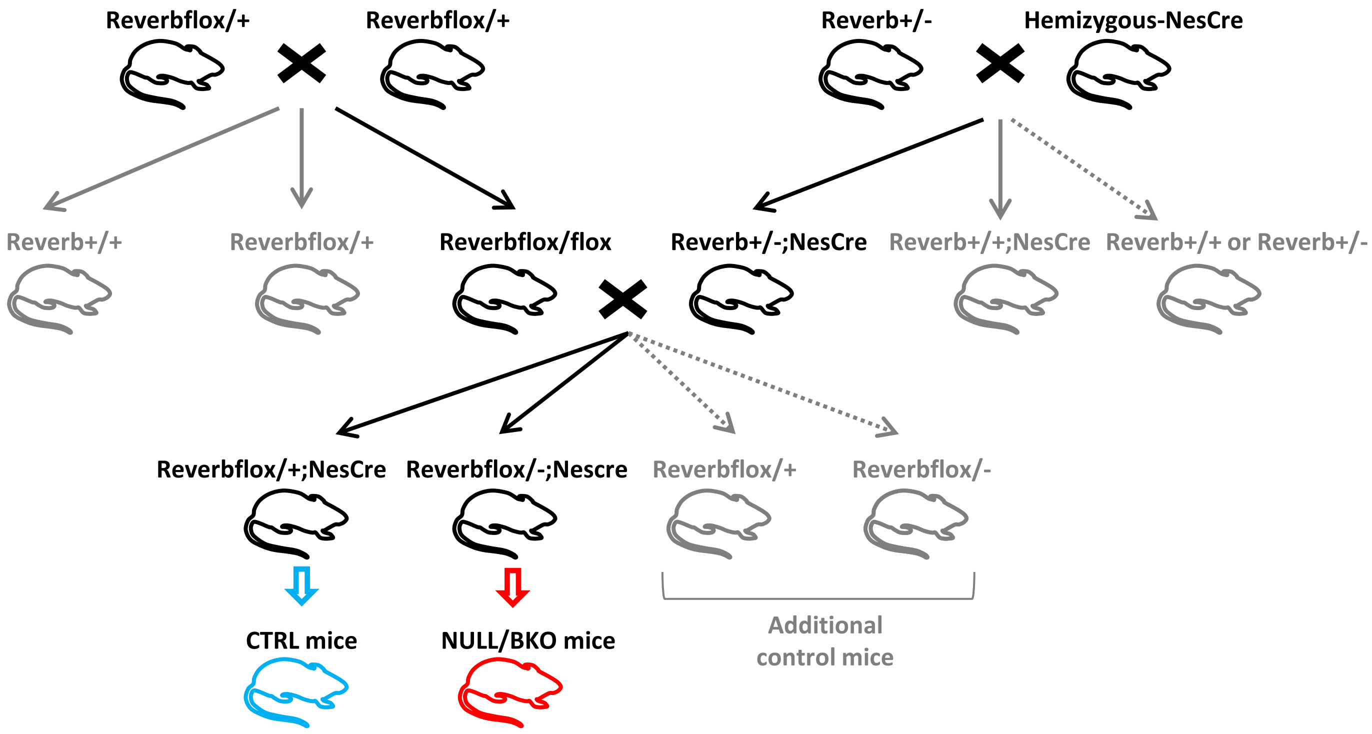

Supplemental tables

Table S1: Cosinor analysis of PER2 expression in the hypothalamus and cerebellum of WT and *Rev-erbα* GKO mice.

Table S2: Cosinor analysis of P-ERK expression in the hypothalamus and cerebellum of WT and *Rev-erbα* GKO mice.

Supplemental Table S1. Cosinor analysis of PER2 expression in the hypothalamus and cerebellum of WT and *Rev-erbα* GKO mice.

		Food *ad libitum*						Restricted feeding					
		WT			GKO			WT			GKO		
		Mean	SEM	P	Mean	SEM	P	Mean	SEM	P	Mean	SEM	P
VMH	a	47.4	3.7	<0.001	*44.6*	*2.5*	*<0.001*	51.5	3.3	<0.001	*53.8*	*3.3*	*<0.001*
	b	18.0	5.2	0.003	*5.6*	*3.5*	*NS*	13.6	4.7	0.01	*6.9*	*4.5*	*NS*
	c	ZT18.6^{a}	1.1	<0.001				ZT11.0^{b}	1.3	<0.001			
ARC	a	52.0	2.5	<0.001	53.2	2.4	<0.001	57.6	3.1	<0.001	53.9	3.7	<0.001
	b	15.7	3.6	<0.001	7.6	3.3	0.04	9.4	4.4	0.05	11.8	5.1	0.04
	c	ZT18.1^{a}	0.8	<0.001	ZT13.2^{ab}	1.8	<0.001	ZT9.8^{b}	1.8	<0.001	ZT10.0^{b}	1.8	<0.001
PVN	a	39.0	2.8	<0.001	38.8	1.6	<0.001	45.1	2.3	<0.001	43.8	2.4	<0.001
	b	10.9	4.0	0.01	4.9	2.3	0.05	10.1	3.2	0.007	9.7	3.5	0.02
	c	ZT17.8^{a}	1.4	<0.001	ZT15.3^{ab}	1.8	<0.001	ZT10.6^{bc}	1.2	<0.001	ZT7.7^{c}	1.3	<0.001
SCN	a	77.9	4.3	<0.001	79.9	3.3	<0.001	84.1	4.8	<0.001	81.8	3.5	<0.001
	b	22.8	6.1	0.001	11.4	4.6	0.03	22.1	6.8	0.001	16.7	4.8	0.004
	c	ZT14.7^{a}	1.0	<0.001	ZT13.6^{a}	1.6	<0.001	ZT10.9^{a}	1.2	<0.001	ZT10.1^{a}	1.2	<0.001
CRB	a	21.7	0.7	<0.001	*22.0*	*1.5*	*<0.001*	*25.1*	*1.5*	*<0.001*	22.4	1.1	<0.001
	b	3.2	0.9	0.003	*2.9*	*2.0*	*NS*	*3.1*	*2.2*	*NS*	4.3	1.7	0.02
	c	ZT19.4^{a}	1.1	<0.001							ZT18.9^{a}	1.3	<0.001

The table S1 shows the three parameters of cosinor regression, including a the mean level, b the amplitude, and c the acrophase of the rhythm (see Methods). For the acrophase, the reference time is Zeitgeber (ZT) 0 (i.e., lights on). NS, non-significant amplitude, making the whole regression not statistically significant (shown by italicized characters; See Methods). For a given structure, the acrophases (c) were compared respectively with t-tests or ANOVA when there were 2 or more groups with significant regressions (c values are not shown when the amplitude was not significant). For a given structure, means lacking common letters are significantly different (p<0.05). WT, wild-type; GKO, global knock-out.

Supplemental Table S2. Cosinor analysis of P-ERK expression in the hypothalamus and cerebellum of WT and *Rev-erbα* GKO mice.

		Food *ad libitum*						Restricted feeding					
		WT			GKO			WT			GKO		
		Mean	SEM	*p*	Mean	SEM	*p*	Mean	SEM	*p*	Mean	SEM	*p*
VMH	a	*146.4*	*5.3*	*<0.001*	*144.6*	*5.2*	*<0.001*	*147.5*	*4.8*	*<0.001*	*144.6*	*5.3*	*<0.001*
	b	*4.0*	*7.5*	*NS*	*14.3*	*7.2*	*NS*	*10.2*	*6.9*	*NS*	*13.4*	*7.5*	*NS*
	c												
ARC	a	166.4	3.8	<0.001	164.1	4.5	<0.001	170.9	2.3	<0.001	*167.5*	*3.4*	*<0.001*
	b	13.8	5.4	0.02	13.9	6.3	0.04	8.5	2.8	0.01	*7.3*	*4.8*	*NS*
	c	ZT13.5[a]	1.5	<0.001	ZT7.2[b]	1.7	<0.001	ZT5.2[b]	1.7	<0.001			
PVN	a	*162.4*	*3.1*	*<0.001*	*165.7*	*3.0*	*<0.001*	161.2	1.9	<0.001	*155.9*	*4.9*	*<0.001*
	b	*6.4*	*4.3*	*NS*	*2.1*	*4.2*	*NS*	5.4	2.4	0.04	*14.5*	*6.9*	*NS*
	c							ZT6.1	2.1	<0.001			
DMH	a	*143.2*	*6.0*	*<0.001*	*146.3*	*4.8*	*<0.001*	*138.4*	*6.8*	*<0.001*	*135.9*	*6.4*	*<0.001*
	b	*12.4*	*8.7*	*NS*	*11.3*	*6.7*	*NS*	*12.6*	*9.0*	*NS*	*18.0*	*9.0*	*NS*
	c												
SCN	a	*160.8*	*4.7*	*<0.001*	*155.4*	*2.3*	*<0.001*	152.1	3.3	<0.001	*146.3*	*4.0*	*<0.001*
	b	*6.8*	*6.4*	*NS*	*5.5*	*3.2*	*NS*	18.6	4.3	<0.001	*9.2*	*5.6*	*NS*
	c							ZT7.8	1.1	<0.001			
CRB	a	50.6	2.9	<0.001	54.1	4.0	<0.001	*57.5*	*3.5*	*<0.001*	*61.5*	*4.1*	*<0.001*
	b	22.0	4.0	<0.001	14.9	5.6	0.02	*6.2*	*5.0*	*NS*	*8.0*	*6.6*	*NS*
	c	ZT23.3[a]	0.7	<0.001	ZT21.5[b]	1.4	<0.001						

The table S2 shows the three parameters of cosinor regression, including a the mean level, b the amplitude, and c the acrophase of the rhythm (see Methods). For the acrophase, the reference time is Zeitgeber (ZT) 0 (i.e., lights on). NS, non-significant amplitude, making the whole regression not statistically significant (shown by italicized characters; See Methods). For a given structure, the acrophases (c) were compared respectively with t-tests or ANOVA when there were 2 or more groups with significant regressions (c values are not shown when the amplitude was not significant). For a given structure, means lacking common letters are significantly different ($p<0.05$). WT, wild-type; GKO, global knock-out.
